# Body-enlarging effect of royal jelly in a non-holometabolous insect species, *Gryllus bimaculatus*

**DOI:** 10.1242/bio.019190

**Published:** 2016-05-16

**Authors:** Atsushi Miyashita, Hayato Kizaki, Kazuhisa Sekimizu, Chikara Kaito

**Affiliations:** Laboratory of Microbiology, Graduate School of Pharmaceutical Sciences, The University of Tokyo, 3-1, 7-chome, Hongo, Bunkyo-ku, Tokyo 113-0033, Japan

**Keywords:** Royal jelly, Body size, Holometabola, Polyneoptera, Silkmoth, Two-spotted cricket

## Abstract

Honeybee royal jelly is reported to have body-enlarging effects in holometabolous insects such as the honeybee, fly and silkmoth, but its effect in non-holometabolous insect species has not yet been examined. The present study confirmed the body-enlarging effect in silkmoths fed an artificial diet instead of mulberry leaves used in the previous literature. Administration of honeybee royal jelly to silkmoth from early larval stage increased the size of female pupae and adult moths, but not larvae (at the late larval stage) or male pupae. We further examined the body-enlarging effect of royal jelly in a non-holometabolous species, the two-spotted cricket *Gryllus bimaculatus*, which belongs to the evolutionarily primitive group Polyneoptera. Administration of royal jelly to *G. bimaculatus* from its early nymph stage enlarged both males and females at the mid-nymph and adult stages. In the cricket, the body parts were uniformly enlarged in both males and females; whereas the enlarged female silkmoths had swollen abdomens. Administration of royal jelly increased the number, but not the size, of eggs loaded in the abdomen of silkmoth females. In addition, fat body cells were enlarged by royal jelly in the silkmoth, but not in the cricket. These findings suggest that the body-enlarging effect of royal jelly is common in non-holometabolous species, *G. bimaculatus*, but it acts in a different manner than in holometabolous species.

## Abstract (precis)

**Summary:** Orally administered royal jelly enlarges non-holometabolous cricket body size, suggesting its conserved effect on body size in insect phyla. This finding contributes to the understanding of regulatory mechanisms on body size.

## INTRODUCTION

The size of living organisms is strictly regulated in each species. Artificial enlargement techniques are useful for elucidating the mechanisms involved in body size regulation, and could contribute to the development of methods to enlarge industrially important organisms. Genetic approaches are currently used to modify animal size, and size-regulating gene mutants have been isolated; however most of the mutants are smaller than wild-type animals, except for limited reports of isolated mutants larger than wild-type animals, e.g. genetic mutants of *Caenorhabditis elegans* that are 1.5-fold larger than the parent worm (Daniels et al., 2000; Hirose et al., 2003). In mice, overexpression of growth hormone enlarges the body twofold compared to parent mice (Palmiter et al., 1982). In addition, mainly in fish and plants, polyploidy causes cell enlargement and results in enlargement of the whole body (Conlon and Raff, 1999; Otto, 2007). These studies have provided significant insight into the principles of size regulation of living organisms, although recent concerns over genetically modified organisms have led researchers to evaluate other types of strategies to enlarge animals for industrial purposes.

As a non-genetic size manipulation, oral ingestion of royal jelly by larvae of the honeybee, *Apis mellifera*, a holometabolous hymenopteran insect, induces queen differentiation, leading to enlarged bodies. Royal jelly contains 12-15% protein, 10-16% sugar, 3-6% lipids (percentages are wet-weight basis), vitamins, salts, and free amino acids (Buttstedt et al., 2014). Royal jelly contains proteins, named major royal jelly proteins (MRJPs), which are associated with queen differentiation (Buttstedt et al., 2014; Huang et al., 2012; Kamakura, 2011). The body-enlarging effect of royal jelly is not only observed in honeybees (Kaftanoglu et al., 2011), but also in silkmoths, *Bombyx mori*, a holometabolous lepidopteran insect (Hayashiya et al., 1965; Nguku et al., 2007). The effect of royal jelly in *Drosophila melanogaster* remains controversial. Kamakura (2011) reported that administration of fresh royal jelly to *D. melanogaster* induces enlargement of body size and fat body cell size, and Kayashima et al. (2012) reported that administration of freeze-dried royal jelly does not enlarge the body size of *D. melanogaster*. The differences in the findings of these two studies may be due to the use of freeze-dried royal jelly in which the responsible substance(s) in royal jelly may be inactivated, but this point remains to be verified. Based on the above studies, we hypothesized that the body-enlarging effect of royal jelly is common in holometabolous insect species. In the present study, we first confirmed this hypothesis using holometabolous silkmoth fed an artificial diet instead of the raw mulberry leaves used in the previous literatures. We also examined whether sex or developmental stage affects the body-enlarging effect of royal jelly in silkmoths.

To expand our knowledge about the body-enlarging effects of royal jelly, we also examined if the size of non-holometabolous species is also affected. As holometabolous insects (characterized by pupation) are evolutionarily more recent, evaluating whether royal jelly has body-enlarging effect in non-holometabolous species provides important information about the universality of the effect in insect phyla. Most contemporary insects belong to Neoptera, comprising Polyneoptera, Paraneoptera, and Holometabola (Misof et al., 2014; Wheeler, 2001), and insects in Polyneoptera and Paraneoptera exhibit hemimetabolous (without pupation) development. The size-regulating system is conserved among insect phyla (Dabour et al., 2011; Edgar, 2006; Lynch et al., 2010), which supports the idea that royal jelly has common effects in holometabolous and non-holometabolous species. In this study, we used the two-spotted cricket *Gryllus bimaculatus*, a Polyneopteran species that goes through hemimetabolous development, to examine whether royal jelly has body-enlarging effects in non-holometabolous species.

## RESULTS

### Effect of royal jelly in *B. mori*

**Fig. 1.**
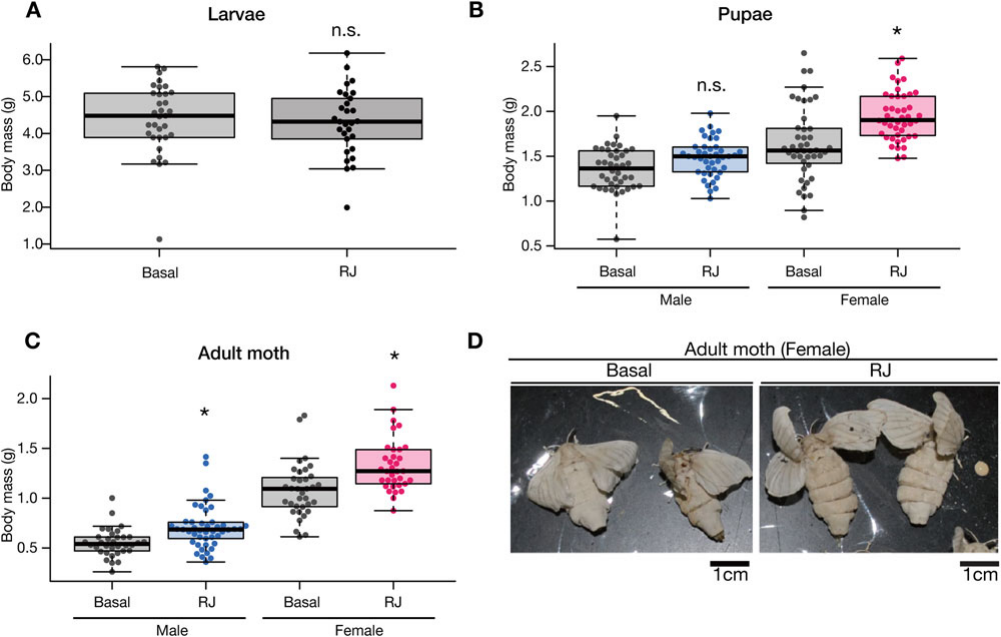
**Effects of royal jelly on silkmoth body size.** (A) First-instar silkmoth larvae were reared by feeding them artificial diets supplemented with 5.6% w/w royal jelly (RJ) or without royal jelly (Basal). The data were obtained on day 23-24 after the start of experiment. The graph shows the pooled data from two independent experiments. All data are plotted in the graph (each dot represents each individual) with boxplots showing quartiles. The statistical information is summarized in Table 1. There was no significant difference (n.s.) between Basal and RJ in the Wilcoxon rank-sum test. (B) First-instar silkmoth larvae were reared to pupae by feeding artificial diets supplemented with 5.6% w/w royal jelly (RJ) or without royal jelly (Basal). The graphs show the pooled data from five independent experiments. Pupae weights were measured on days 31-40 after the start of experiment. In each experiment, larvae were fed the diets throughout larval stage (pupae and adults do not ingest any diet). All data are plotted in the graph (each dot represents each individual) with boxplots showing quartiles. Asterisks indicate a significant difference (*P<0.025) compared to the Basal diet group in the Wilcoxon rank-sum test. The statistical information is summarized in Table 1. (C) First instar silkmoth larvae were reared to adult moths by feeding artificial diets supplemented with 5.6% w/w royal jelly (RJ) or without royal jelly (Basal) throughout larval stage. Weights of adult moths were measured on the day of adult emergence from pupae. The graph shows the pooled data from five independent experiments. All data are plotted in the graph (each dot represents each individual) with boxplots showing quartiles. Asterisks indicate a significant difference (*P<0.025) compared to the Basal diet group in the Wilcoxon rank-sum test. The statistical information is summarized in Table 1. (D) An image of female moths reared by feeding on an artificial diet supplemented with 5.6% w/w royal jelly (RJ) or without royal jelly (Basal). Scale bar: 1 cm.

First, we confirmed that royal jelly enlarged body size in silkmoth *B. mori* under our experimental conditions, in which we fed silkworms an artificial diet instead of raw mulberry leaves. As a result we observed royal jelly-induced body enlargement in female pupae and adults, but not in larvae or male pupae (Fig. 1A-C, Table 1), and adult female moths administered royal jelly exhibited swollen abdomens (Fig. 1D). These findings confirmed that royal jelly enlarges body size in *B. mori* under these conditions and suggest that the effect of royal jelly depends on the developmental stage and sex.

**Table 1.**
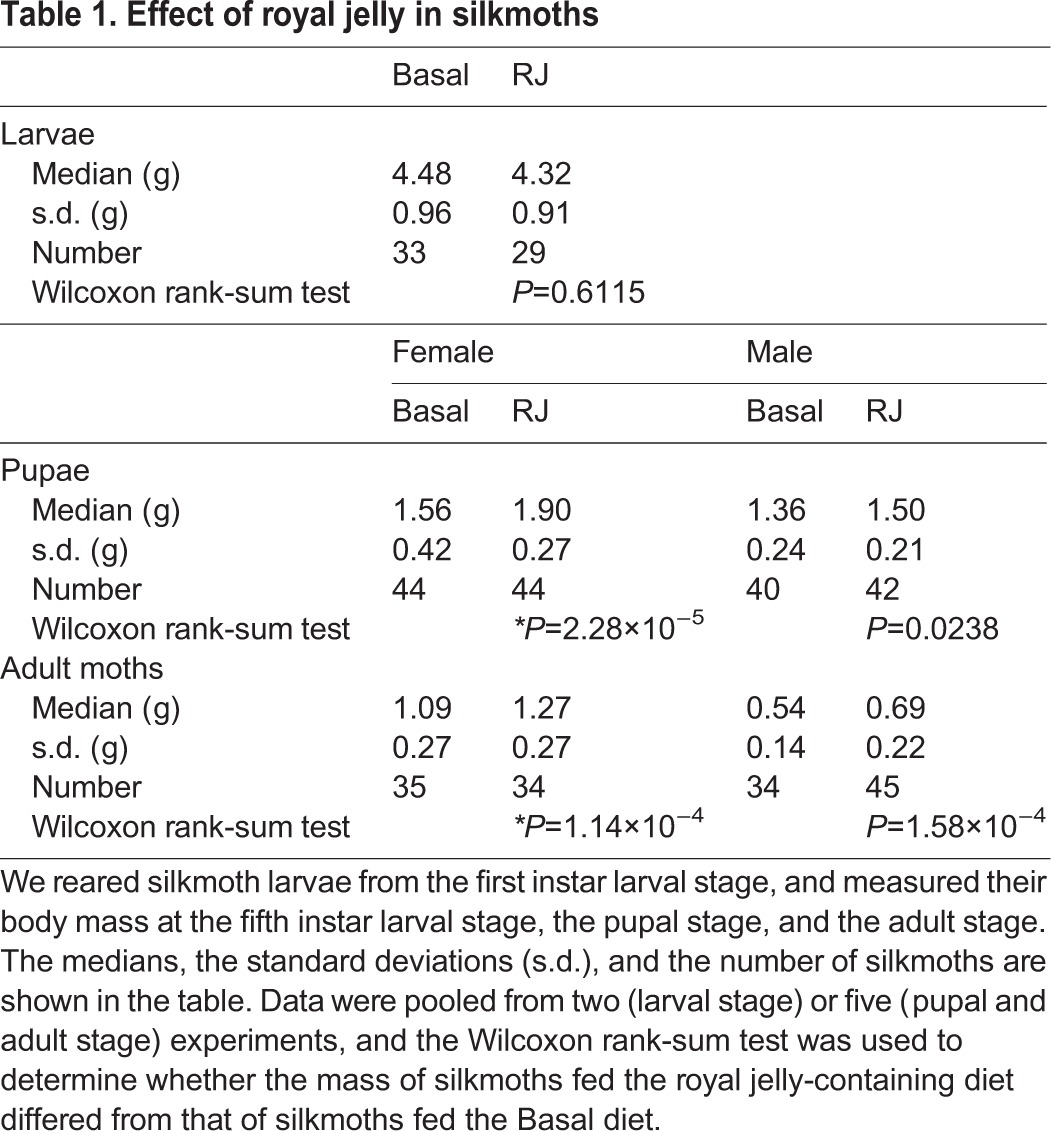
**Effect of royal jelly in silkmoths**

### Effect of royal jelly in *G. bimaculatus*

**Fig. 2.**
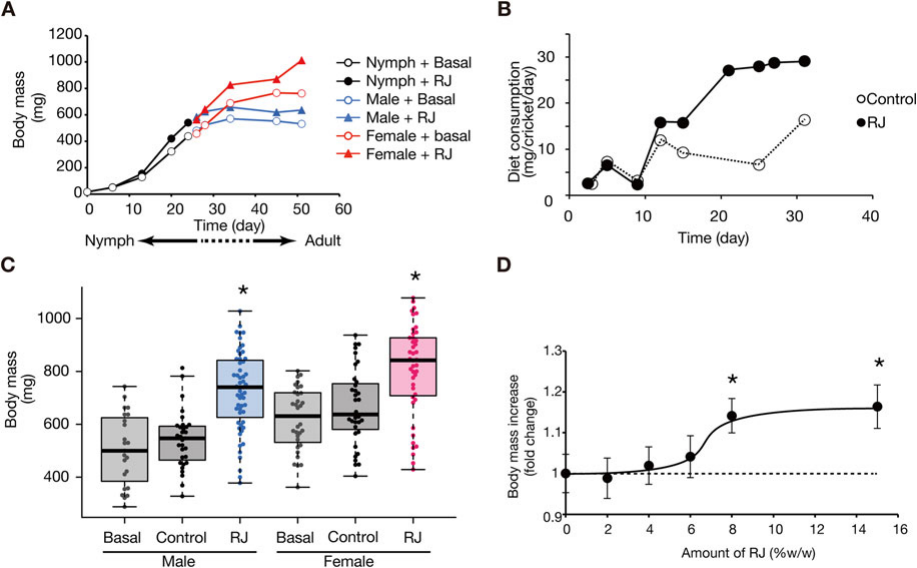
**Effects of royal jelly on cricket body size.** (A) Crickets (*n*=86-94) were reared by feeding on artificial diets supplemented with 15% w/w royal jelly (RJ) or without royal jelly (Basal) from the second instar larval stage. The vertical axis represents cricket weight and the horizontal axis represents time after the start day of the experiment. Mean cricket weights are shown in the graph. The weights of crickets in the early and middle nymph stage, during which males and females cannot be distinguished by their appearance, are indicated by black-filled circles (RJ) and open black circles (Basal), respectively. After the late nymph stage, when cricket sex can be distinguished by the presence of the ovipositor, male weights are shown in blue and female weights are shown in red. Filled triangles indicate the weights of crickets on the RJ diet, and open circles indicate those of crickets on the Basal diet. (B) Diet consumption (mg diet/cricket/day) of the crickets (*n*=20-128) was measured. The diet consumption at variable periods is indicated by the black-filled circles (RJ) and open black circles (Control). Data from three independent experiments were pooled. (C) Cricket nymphs reared by feeding a Basal diet (Basal), the RJ diet (RJ), Control diet (Control) from the second instar nymph stage. Cricket weights of male adults or female adults were measured on the day of final molt (*n*=23-55). The graph shows the pooled data from three independent experiments. All data are plotted in the graph (each dot represents each individual) with boxplots showing quartiles. Asterisks indicate a significant difference (*P<0.025) compared to the Basal diet group in the Wilcoxon rank-sum test. The statistical information is summarized in Table 2. (D) Second instar cricket nymphs were reared by feeding diets containing 0, 2, 4, 6, 8, or 15% w/w RJ. Body weights were measured at day 14 (*n*=28-32/dose). Means±s.e.m. values are shown, *P<0.05 by Student's *t*-test of the mean compared to Basal diet (0% RJ).

To examine whether royal jelly enlarges non-holometabolous species, we orally administered royal jelly to nymphs of cricket *G. bimaculatus*. We fed the crickets a royal jelly-containing diet from their early nymph stage, and measured their body weight at several time-points after starting royal jelly administration. Increased body mass was observed after the mid-nymph stage in both males and females (Fig. 2A). This finding suggests that the body-enlarging effect of royal jelly is common in insect phyla, including non-holometabolous species, although the response of crickets to royal jelly differed from that of *B. mori* in terms of the developmental period in which the effect is observed. As for food consumption (per cricket), crickets fed the royal jelly diet consumed more food than crickets fed the control diet at the nymph stage (Fig. 2B). This finding indicates that the royal jelly affects cricket size via upregulation of food consumption.

We further examined whether the effect of royal jelly can be explained by changes in the protein, lipid, and carbohydrate composition of the diet. Administration of diets supplemented with a comparable amount of proteins, lipids, and carbohydrates found in royal jelly (control diet) did not increase body weight (Fig. 2C, Table 2). The result indicates that the effect of royal jelly was not simply due to an increased amount of protein, sugar, and lipid in the diet. Also, the effect of royal jelly was dose-dependent (Fig. 2D), in which 8 % w/w or 15 % w/w royal jelly exhibited a body-enlarging effect.

**Table 2.**
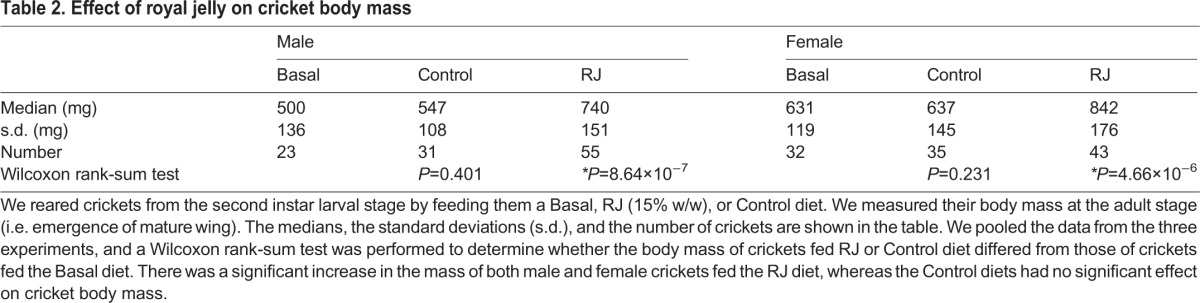
**Effect of royal jelly on cricket body mass**

### Royal jelly uniformly enlarges the whole body of *G. bimaculatus*

**Fig. 3.**
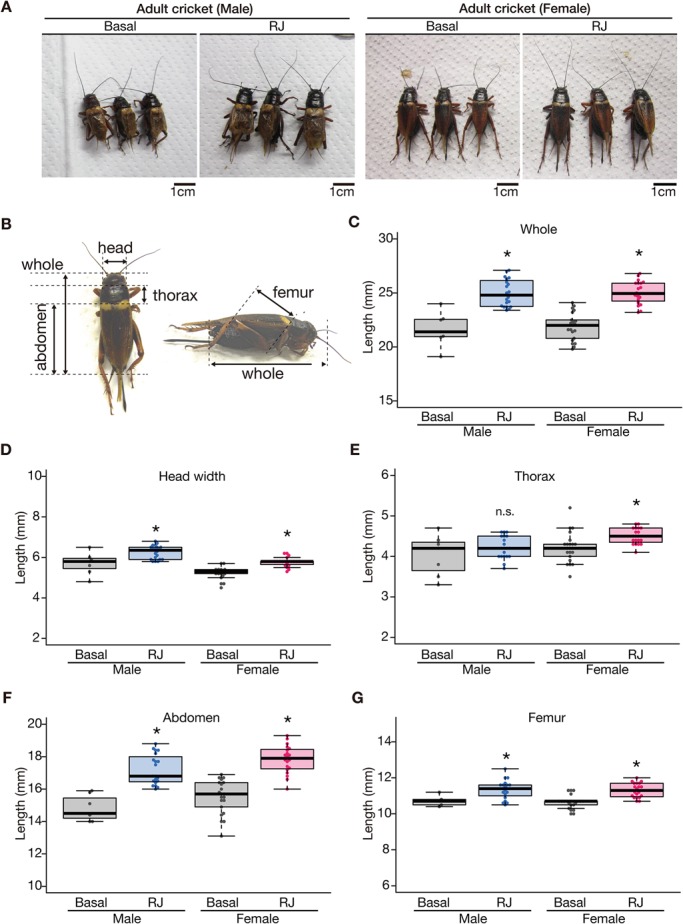
**Enlargement of cricket body parts by feeding on royal jelly.** (A) Cricket nymphs at second instar nymph stage were reared to adults with the Basal diet (Basal), control diet (Control) or royal jelly diet (RJ). Images were obtained on the day of the final molt. Scale bar: 1 cm. (B) Lengths of the body parts we measured in this study are shown. A female cricket is shown in the panel with arrows, indicating the length measured. (C-G) Lengths of body parts are shown. The vertical axes indicate the length of cricket body parts (mm). Crickets were grouped by sex, and the results from those fed the Basal diet (Basal) and royal jelly diet (RJ) are indicated. Asterisks indicate a significant difference (*P<0.025) compared to the Basal diet group in the Wilcoxon rank-sum test. The data and statistical information are summarized in Table 3.

We then examined whether the body-enlarging effect of royal jelly is specific among body parts. Based on simple observation, royal jelly administration did not enlarge a specific body area, but rather uniformly enlarged the whole body (Fig. 3A). To quantitatively confirm this observation in crickets, we measured the sizes of body parts of royal jelly-fed or basal diet-fed crickets. We measured the lengths of the whole body (from the top of the head to the tip of the abdomen), thorax, abdomen, femur, and the width of the head (Fig. 3B). Every part of the cricket body examined, except the male thorax, was enlarged by royal jelly (Fig. 3C-G, Table 3).

**Table 3.**
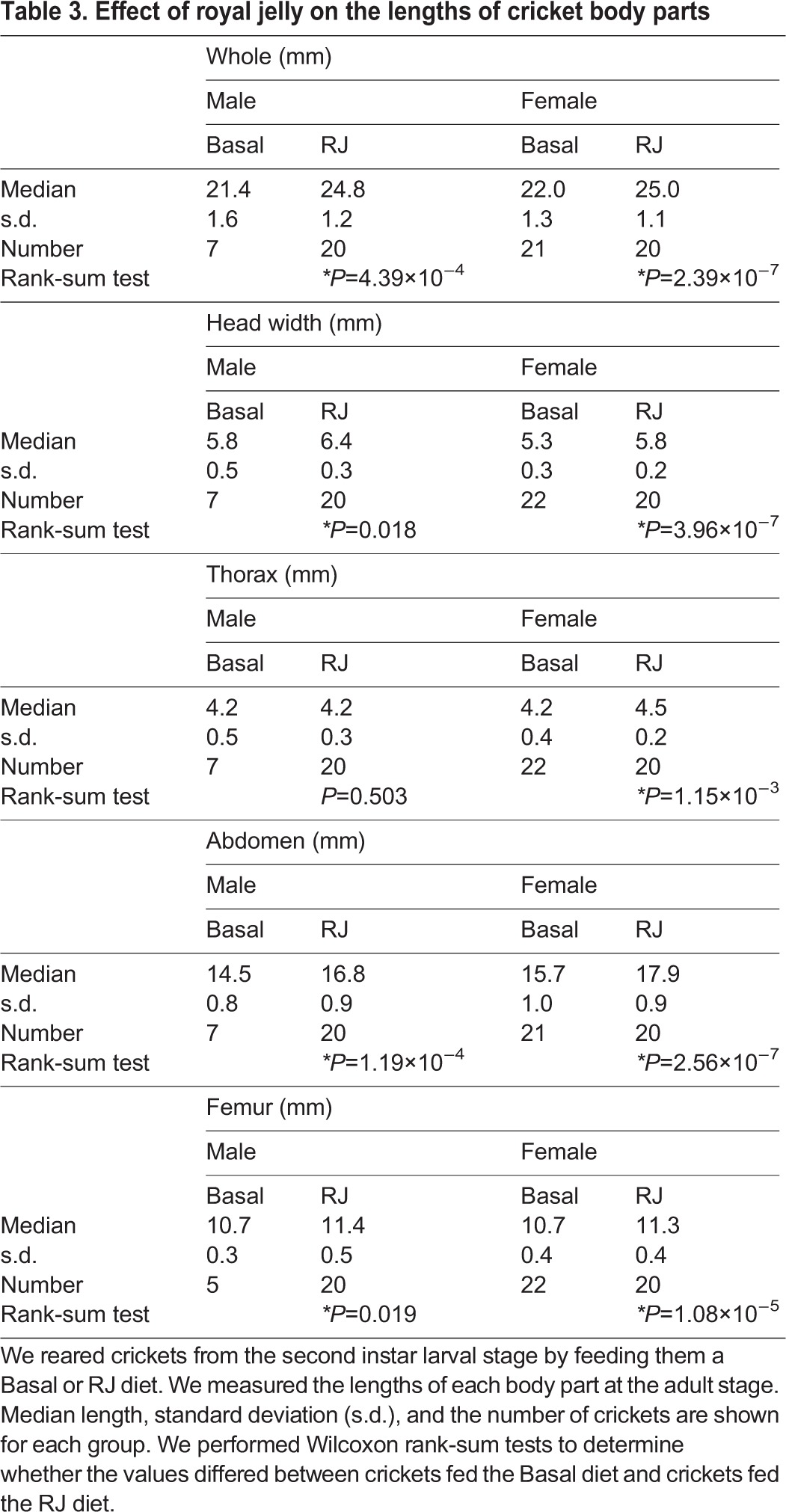
**Effect of royal jelly on the lengths of cricket body parts**

### Effect of royal jelly on development time and longevity of *G. bimaculatus*

Royal jelly is reported to extend the longevity of nematodes (Detienne et al., 2014; Honda et al., 2011) and fruit flies (Gardner, 1948). We examined whether royal jelly has a similar effect on *G. bimaculatus* longevity. Crickets fed royal jelly survived longer than those fed the basal diet (Fig. S1A, Table S1), indicating that the positive effect of royal jelly on lifespan is conserved in Polyneoptera. The adults emerged earlier among crickets fed the royal jelly-containing diet than among crickets fed the basal diet (Fig. S1B). Thus, the prolonged lifespan in the royal jelly-fed crickets was not due to an extended nymph stage.

### Effect of royal jelly on cell size in *B. mori* and in *G. bimaculatus*

**Fig. 4.**
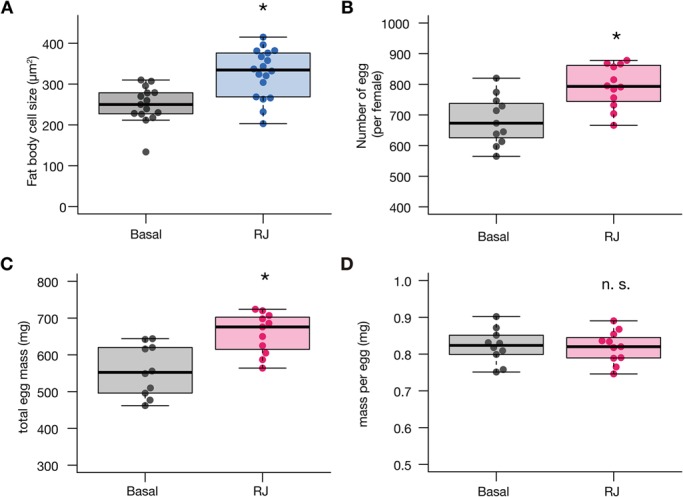
**Effect of royal jelly on silkmoth fat body cells and eggs.** (A) First instar silkmoth larvae were reared to adult moths by feeding artificial diets supplemented with 5.6% w/w royal jelly (RJ) or without royal jelly (Basal) throughout the larval stage. Fat body cells from male silkmoths were observed by microscopy and the cell area was measured as described in the Materials and Methods. The vertical axis indicates the cell area (µm^2^). All data are plotted (each dot represents each individual) in the graph with boxplots showing quartiles. Asterisk indicates a significant difference compared to the Basal diet group in the Wilcoxon rank-sum test (*P*=5.36×10^−4^). (B-D) Eggs were collected from the silkmoth abdomen on the day of adult emergence, and the number (B), total mass (C), and mass per egg (D) were calculated. All data are plotted (each dot represents each individual) in the graph with boxplots showing quartiles. Asterisk indicates a significant difference compared to the Basal diet group in the Wilcoxon rank-sum test (B, *P*=3.56×10^−3^; C, *P*=8.33×10^−3^). There was no significant difference (n.s.) between the two groups in mass per egg (D, *P*=0.973).

**Fig. 5.**
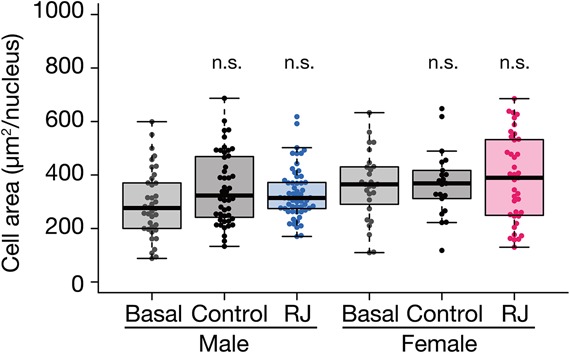
**Effect of royal jelly on cricket fat body cells.** Cricket nymphs at second instar nymph stage were reared to adult with the Basal diet (Basal), control diet (Control), or royal jelly diet (RJ). Fat body cells from crickets were observed by microscopy and the cell area was measured as described in the Materials and Methods. The vertical axis indicates the cell area (µm^2^). All data are plotted (each plot represent each individual) in the graph with boxplots showing quartiles (outliers are plotted as individual points). Control diet and RJ diet-fed males and females were not significantly different (n.s.) from Basal diet-fed males and females.

In *D. melanogaster*, royal jelly increases the size of fat body cells (Kamakura, 2011). To examine whether the body-enlarging effect of royal jelly on fat body cells is conserved in *B. mori* and *G. bimaculatus*, we prepared sliced specimens and performed microscopic observations of the fat body. In silkworms, royal jelly enlarged the fat body cells (Fig. 4A). The number and total mass of eggs loaded in the female abdomen on the first day of the final molt were also increased (Fig. 4B,C), whereas the mass of each egg remained comparable to those fed the control diet (Fig. 4D). In contrast, we did not observe such an increase in the fat body cell size in crickets (Fig. 5). We did not analyze the effect of royal jelly on cricket eggs, as cricket adults, unlike silkmoths, do not contain mature eggs loaded in the abdomen on the day of the final molt.

## DISCUSSION

The present study demonstrated that royal jelly has a body-enlarging effect on a non-holometabolous species, *G. bimaculatus*, which belongs to Polyneoptera. This is the first report of the body-enlarging effect of royal jelly in Polyneopteran insects, a more primitive group than Holometabola. Histologic analysis revealed the differential action of royal jelly on cell number/size regulation depending on cell type and species; based on the comparison between *B. mori* and *G. bimaculatus*, peroral administration of royal jelly increases body size via different mechanisms in the two species which may reflect a difference in the developmental processes between holometabolous and non-holometabolous species. To further investigate the effect of royal jelly from an evolutionary point of view, it would be interesting to examine whether royal jelly affects the size of more primitive insect species in Palaeoptera, such as dragonflies.

Holometabolous insects such as bees, flies and moths have evolutionarily acquired a developmental system with a pupal stage, holometabolism. In contrast, Polyneopteran insects, such as crickets, grasshoppers, cockroaches, and termites, appeared earlier than holometabolous species and have retained hemimetabolism, a developmental process that lacks a pupal stage (Wheeler, 2001). The findings of the present study revealed that royal jelly enlarges the Polyneopteran insect *G. bimaculatus*, which suggests that insects with hemimetabolous developmental processes have mechanisms that respond to royal jelly to induce the enlargement, just as in holometabolous insects. Thus, the mechanism that recognizes royal jelly to enlarge the body may not be acquired specifically in holometabola, but is more likely to have been acquired earlier in the origins of Neoptera. Considering that silkmoths and crickets seldom eat royal jelly in their natural environments, we speculate that a particular molecule in royal jelly mimics signaling molecules in silkmoths and crickets that have a positive role in their size regulation. Factors controlling body size, such as insulin receptors, p70 ribosomal S6 kinase, and epidermal growth factor receptors, are conserved between holometabolous insects and polyneopteran insects (Dabour et al., 2011; Edgar, 2006; Lynch et al., 2010). We speculate that the mechanism by which royal jelly acts in silkmoths and crickets is associated with conserved pathways such as epidermal growth factor pathways, although some other pathway(s) could be responsible for the difference in the effect of royal jelly on fat body cell size between silkmoths and crickets.

The sex specificity of the effect of royal jelly differed between silkmoths and crickets. In *B. mori*, the effect of royal jelly to enlarge body size was only observed in female pupae and adult moths (Fig. 1B,C). As ovary maturation in *B. mori* occurs at the pupal stage and the ovary occupies ∼50-60% of the body mass, it is likely that the effect of royal jelly appears in females earlier than in males. In contrast, ovary maturation in crickets occurs gradually after adult emergence, so it is reasonable to deduce that the sexual dimorphism in body mass affected by royal jelly is relatively milder in crickets at the early adult stage.

This study did not identify the responsible molecule(s) in royal jelly that enlarges crickets and silkmoths. The increased amount of proteins, sugars, and lipids in the diet did not account for the enlargement (Fig. 2C), but we cannot exclude the nutritional effects of royal jelly that enlarge insect bodies, as a variety of nutritional substances, such as vitamins, are present in royal jelly. At least two possibilities remain for the body-enlarging effect of royal jelly: royal jelly initially promotes food intake to confer an enlarged body, and royal jelly affects body size by a specific signaling mechanism and food intake is increased as a result. The determinants in royal jelly to induce queen differentiation in honeybees are controversial between MRJP1 (Kamakura, 2011) or MRJP3 (Huang et al., 2012), which are abundant proteins in royal jelly, and other factors, including 10-hydroxy-2-decenoic-acid (Spannhoff et al., 2011) or sugar composition (Asencot and Lensky, 1988; Buttstedt et al., 2014). A recent report demonstrated that 10-hydroxy-2-decenoic-acid extends the life span of *C. elegans* (Honda et al., 2015). In the present study, we purified MRJP1 (Fig. S2), but it did not have body-enlarging activity in crickets (A.M., H.K., K.S. and C.K., unpublished data). Identification of the responsible molecule(s) in royal jelly to enlarge cricket and silkmoth size requires further investigation.

## MATERIALS AND METHODS

### Insects, diets and rearing

#### Silkmoths

Silkmoth *Bombyx mori* eggs (Fu/Yo×Tsukuba/Ne) were purchased from Ehime-Sanshu (Ehime, Japan). Hatched larvae were fed an artificial diet, ‘Silkmate’ (Nihon Nosan, Kanagawa, Japan), in an incubator at 27°C. To prepare the royal jelly diet for silkmoths, we mixed royal jelly with Silkmate so that the final royal jelly content was 5.6 % w/w (dry weight basis). The dose was determined by an experiment using different doses of royal jelly in the diet.

#### Crickets

Cricket *Gryllus bimaculatus* nymphs were obtained from Tsukiyono-Farm (Gunma, Japan). We collected fertilized eggs, and hatched nymphs were used for the experiments. Hatched nymphs were fed cricket basal (Basal) diet ‘Koorogi-food’ (Tsukiyono-Farm) in an incubator at 28°C. The Basal diet contained 30% corn, 30% soybeans, 25% rice bran, 10% fish meal, and 5% mulberry powder (dry weight basis). For crickets, adequate amounts of water were absorbed in Kimtowels and placed in the cage. Crickets were kept in a cage as a group. A 12-h light/dark cycle was maintained. To prepare the royal jelly diet for the crickets, we mixed royal jelly with the cricket diet so that the final royal jelly content was 15 % w/w (dry weight basis). The dose was determined by an experiment using different doses of royal jelly in the diet (shown in Fig. 2D). The control diet contained comparable amounts of protein, sugar, and lipid, and was prepared according to a previous report (Kamakura, 2011).

### Royal jelly

Honeybee royal jelly was purchased from Kumagaya-Yoho (Saitama, Japan), and stored at 4°C.

### Measurement of insect body weight and size

To measure the body weights of silkworms, silkmoth pupae, and silkmoths, we captured them individually in a weighing bowl. Net weights were measured using an electronic balance (Shimadzu EB-430D, or Mettler Toledo AB54-S). To measure cricket body weights, we captured each live cricket individually in a 50-ml sterile polypropylene tube and measured the net weight. To measure the lengths of cricket body parts, we froze the crickets at −20°C. The lengths were measured using a Vernier caliper. Data were pooled and analyzed using the Wilcoxon rank-sum tests or Student's *t*-tests. Data are summarized in Table 1-4. Statistical analysis was performed using R ver. 3.2.1 (R Foundation for Statistical Computing, Vienna, Austria) running on Mac OS X for Wilcoxon rank-sum tests, and Microsoft Excel 2011 for Student's *t*-tests.

### Calculation of diet consumption

The diet was placed in a plastic tray, and the initial dry weight was determined. After each feeding interval, the dry weight of the food remaining in the tray, from which feces were removed using tweezers to the extent possible, was measured. The net consumption per cricket was then calculated according to the following formula:
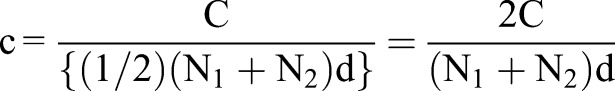


c, diet consumption per cricket (mg diet/cricket/day); C, total net consumption (experimentally measured) (mg); N_1_, total cricket number at the start of the feeding interval; N_2_, total cricket number at the end of the feeding interval; d, length of feeding interval (days). Over the course of the experiment, dead crickets were removed and not included in the cricket number for analysis.

### Measurement of tissue cell size

#### Eggs

To obtain silkmoth eggs, we dissected adult female silkmoths (on the day of adult emergence) and scraped eggs out of the abdomen. The total mass of eggs was then measured, and then suspended in tap water to detangle the oviducts. The number of eggs was counted for each silkmoth. Mass per egg was determined by dividing the total mass by the number of eggs.

#### Fat body cells

To obtain fat body cells from both silkmoths and crickets, we dissected adult individuals on the first day of adult emergence, and scraped out the fat body tissues (white abundant tissues present in the abdomen of male silkmoths and of both sexes in crickets), and stored them in 4% paraformaldehyde/phosphate-buffered saline (pH 7) at 4°C for more than 48 h. The fixed samples were then suspended in 70%, 80%, 90%, 95%, 100% (dehydrated); 100% (dehydrated) ethanol; 100% xylene; 100% xylene sequentially for at least overnight in each step. After suspending the samples in xylene, they were then suspended in melted paraffin at 70°C for 24 h. The paraffin block (solidified at room temperature) was then sliced using a microtome (Leica, SM2000R, thickness=0.5 µm). The sliced specimens were dried at 43°C, and stained with a Meyer's hematoxylin solution and 0.25% eosin solution. We performed microscopic observation of the stained tissues and took photos of the fat body tissues from each individual. Cell area was measured using ImageJ (NIH) (scales were adjusted using the ‘calibrate’ command, and areas were measured using the ‘measure’ command). In silkmoths, only males contained a detectable amount of fat body, and cells in the fat body exhibited clear borders for each cell. Thus, we measured the areas of each single cell in silkmoths. In contrast, both male and female crickets contained enough fat body and the cell borders were subtle; thus we measured total area of fat body tissue counting the number of nuclei to determine the cell area (dividing the total area by number of nuclei).

### Evaluation of cricket lifespan

Cricket survival was monitored by counting the number of crickets in the cage at different time-points. Survival of crickets fed royal jelly (15% w/w) or the Basal diet was observed in two independent experiments. The pooled data were used to perform a log-rank test to evaluate the difference in the survival curves between crickets fed the diet with or without royal jelly. The experiment was censored at 85 days. The log-rank test was performed using R for Mac OS X, ver. 2.5.3, using the ‘survival’ package.

### Purification of MRJP1 from royal jelly

We suspended 1 g of royal jelly in 33 ml of 20 mM Tris-HCl pH 7 on ice and centrifuged the mixture (2000×***g***, 30 min, 4°C). The pellet was then suspended in 5 ml of the same buffer on ice and centrifuged (2000×***g***, 30 min, 4°C); this step was sequentially repeated four times and each supernatant fraction was stored at 4°C. The royal jelly, supernatant fractions (six fractions), and final pellet were analyzed by sodium dodecyl sulfate polyacrylamide gel electrophoresis on 12.5% sodium dodecyl sulfate-polyacrylamide gels, and protein bands were stained by Coomassie Brilliant Blue. Fractions from 3 to 6 were pooled and dialyzed against 20 mM Tris-HCl buffer (pH 7).
